# Concentrated Remedies

**Published:** 1868-09

**Authors:** F. R. Payne

**Affiliations:** Marshall, Ill.


ARTICLE XXXIV.

### CONCENTRATED REMEDIES.

By F. R. PAYNE, M.D., Marshall, Ill.

Read before the Clark County Medical Society, July 1st, 1868. Published by request of the Association.

It is our purpose in this paper to briefly refer to some of the organic remedies which, by long experience, we have found valuable aids in assisting nature in the removal of disease. It should always be borne in mind by the physician, that nature tends, in a majority of cases, to repair injuries inflicted upon the body, and throw off morbid or deranged conditions of the system. This instinctive power, if properly aided by a judicious and rational system of hygiene, often effects the cure, especially in those of a mild character. If a disease presents itself for treatment it is the duty, and demanded of all honest and honorable physicians, to inquire and satisfy themselves whether or not hygienic means are capable of accomplishing the objects desired; if so, they should always be employed in preference to medicine. We too often ascribe the credit to some remedy, when nature performed the cure.

It is my purpose, on this occasion, to present some remedial agents which we regard as worthy of favorable consideration.

### ACONITIN.

This is derived from the *Aconitum Napellus*, or wolfsbane, and is contained in both the leaves and roots. It has three principles: resin, neutral, and alkaloid. Its properties are diaphoretic, diuretic, alterative, antispasmodic, and narcotic; consequently we can, with at least a fair prospect of benefit, employ it in the treatment of phthisis, dropsy, gout, rheumatism, and nearly all forms of nervous disorder.

When given in small and repeated doses, it excites diaphoresis and diuresis, and increases many of the secretions. Its continued use for a long time may produce exanthematic eruptions and itching, and, frequently, pain in the joints. In overdoses, it will produce paralysis of the tongue and pharynx, suffocation, vomiting, diarrhœa, convulsions, and death. The dynamical effects of this remedy show conclusively that it is a stimulant to the nervous system.

These physiological influences of aconitin make it a valuable agent in arousing peripheric secretions, chronic diseases of the sheaths of muscles, nerves, and tendons.

We have found either aconitin or alcoholic tincture of aconite leaves a valuable arterial sedative; it is in the latter form we usually prescribe the remedy. We have given it with marked benefit in some cases of epilepsy and neuralgia; it is a valuable local remedy in tic-douloureux. The dose of aconitin is from $\frac{1}{24}$ to $\frac{1}{12}$ of a grain, and the tincture 6 to 10 drops every 4 or 6 hours.

XANTHOXYLIN.

This remedy is obtained from the *Xanthoxylum Fraxineum*, or prickly ash. The part used is the bark, and contains a resinoid and neutral principle. Its properties are stimulant, alterative, sialogogue, and, to some extent, tonic. It may be employed in rheumatism, colic, indigestion, paralysis, etc.

We have used this agent extensively, and have great confidence in its value as a remedy in the above diseases. It is true that we generally use it in combination with other appropriate agents. It is a valuable medicine in arousing the activity of the muscular fibres of the stomach and bowels, and excites an increased flow of the gastric and digestive juices, and thus aids in establishing normal action of the mucous surfaces. We are perfectly satisfied, by long experience, that it is a valuable addition, and gives additional permanency to the remedial influences of other tonics and stimulants. The active principle may be given in from 2 to 4 grains.

In combination with fraserin, we have found it valuable in chronic diarrhœa and improving cases of dysentery. We frequently use the bark, but it is much more convenient to have the active principle, provided always we can get that which contains all of the medicinal properties of the bark.

FRASERIN.

This remedial agent is derived from the *Frasera Carolinensis*, or American colombo. The part used is the root, and it contains, by chemical analysis, a resin, neutral, and muci-resin. The properties of this remedy are, tonic, stimulant, and slightly astringent.

We have employed it with marked benefit in many cases of indigestion, debility, and chronic dysentery; but the mucous membranes seem to be especially benefited by the use of this remedy. We have used it with marked benefit in weakness of the digestive organs, and in all disorders resulting from abnormal action of the digestive apparatus. We frequently administer it in combination with *gentiana ochroleuca*, with decided benefit in all derangements of the digestive apparatus. The dose of the active principle is from 3 to 8 grains, but the powdered root may be administered in from 10 to 30 grains.

PRUNIN.

This medicinal agent is found in nearly all parts of the United States. The prunin is obtained from the *Prunus Virginiana*, or wild cherry. The part used is the bark, and it contains a resinoid, neutral, and amygdalin. The properties of this remedy are stimulant, tonic, expectorant, and sedative. The seeds of this tree contain prussic acid, and are beneficial in colds, coughs, incipient phthisis, and bronchial affections attended with severe cough.

The prunin may be given with every hope of success in ordinary cases of debility caused by cold and protracted cough. In those cases where there is a tuberculous tendency, bitter tonics are liable to impair the appetite, and thus favor disintegration; but prunin, or even the bark or seed of the wild cherry, may be administered without fear of nauseating the stomach. It contains a peculiar principle that promotes the appetite, strengthens digestion, and quiets spasmodic cough by allaying the irritability of the nervous system. It calms inordinate action of the heart and arterial system. When we cannot conveniently procure the prunin, we recommend the use of the cherry-seed, bruised and put into a sufficient quantity of spirits to preserve them. We have, in some cases where there is constriction of the chest and dyspnœa, found it beneficial to combine prunin with eupatorin, veratrin, and other antispasmodics and expectorants. The dose of pure prunin is from 2 to 8 grains. It is of vast importance, in using these concentrated remedies, that we procure pure and genuine articles.

HYOSCYAMIN.

This is said to contain all the medicinal virtues of the *Hyoscyamus Niger*, or henbane. Its properties are anodyne, antispasmodic, soporific, narcotic, diuretic, and laxative.

We have frequently used this remedy in neuralgia, pertussis, bronchitis, and all catarrhal and nervous affections. We regard it, in combination with belladonin and podophyllin, as a superior remedy in costiveness, and many forms of female diseases, where the nervous system is implicated. Our usual mode of administration is in a small pill, and we proportion each article according to its properties and physiological effects and the peculiarity of the disease. We have very seldom prescribed this pill without future calls for the same medicine. Great care should be taken in the quantity of atropin.

The various officinal preparations of this plant are often uncertain. When a genuine article is procured, we may confidently expect beneficial results in the treatment of delirium, puerperal insanity, after-pains, cough and dyspnœa, neuralgia, rheumatism, irritable ulcers, and hemorrhoids. In hyperæsthesis of the nervous system, acute morbid sensibility of the organs of special sense, this remedy will be found invaluable. The dose is ¼ to 1 grain.

ATROPIN.

This is derived from the *Atropia Belladonna*, or deadly nightshade. Its properties are narcotic, anodyne, calmative, antispasmodic, alterative, resolvent, and diuretic. We have employed it in epilepsy, convulsions, scirrhus, pertussis, costiveness, and nervous affections generally, with decided benefit. The dynamical effects of this remedy when given in small doses are, thirst, dryness of the fauces, difficult deglutition, deluded vision. When given in overdoses, alkalies, especially liquor potassæ, is a good antidote.

It seems to be pretty well established that its chief physiological effects and therapeutic use, as an internal remedy, depend upon the decided influence it exerts upon the pneumogastric nerve. If we cannot procure a reliable preparation of atropin, we use the solid extract in from $\frac{1}{6}$ to $\frac{1}{2}$ grain doses; the proper dose of atropin being from $\frac{1}{24}$ to $\frac{1}{12}$ of a grain.

We have no hesitancy in recommending this remedial agent as one of superior value in the diseases above enumerated. The important indications in the employment of this remedy are in the formative stages of organic affections. It has a decided influence in overcoming the abnormal sensibility which produces and accompanies structural change. The use of other stimulating resolvents, not possessing the auxiliary properties of this, would, in all probability, increase the irritation and thus favor structural lesion. We have used it with decided advantage in the prevention of abortion resulting from increased sensibility and contractility of the uterus.

We generally combine it with other appropriate remedial agents. It is a powerful remedy, and should be used with great care, especially in children; and, in fact, when given in large but not poisonous doses to adults, it produces extensive dilatation of the pupils, and alarms the patient. This effect is often produced by the local application of the remedy.

It is my intention, at our future meetings, to continue the presentation of remedies, both organic and mineral, which we have found beneficial in assisting nature in the cure of disease; and we would be pleased to have the members of this Society, at each meeting, give their experience for or against the various remedies now used in practice.

Therapeutics, which means "I cure," is a very important department of our profession, but it is intimately connected with pathology. A thorough knowledge of disease is indispensably necessary if we hope to be successful therapeutists.

